# Modulation of Human Colon Cell Activity by Synthetic Coumarin Derivatives Bearing a Phosphonate Group

**DOI:** 10.3390/molecules30132846

**Published:** 2025-07-03

**Authors:** Katarzyna Szwaczko, Roman Paduch, Kamil Dziuba, Krzysztof Szafrański, Adrian Wiater

**Affiliations:** 1 Department of Organic Chemistry and Crystallochemistry, Institute of Chemical Sciences, Faculty of Chemistry, Marie Curie-Skłodowska University, Gliniana 33, 20-614 Lublin, Poland; katarzyna.szwaczko@mail.umcs.pl (K.S.); kamil.dziuba@mail.umcs.pl (K.D.); 2 Department of Virology and Immunology, Institute of Biological Sciences, Faculty of Biology and Biotechnology, Maria Curie-Skłodowska University, Akademicka 19, 20-033 Lublin, Poland; 3 Department of General and Pediatric Ophthalmology, Medical University of Lublin, Chmielna 1 Street, 20-079 Lublin, Poland; 4 Department of Organic Chemistry, Medical University of Gdańsk, Al. Gen. J. Hallera 107, 80-416 Gdańsk, Poland; krzysztof.szafranski@gumed.edu.pl; 5 Department of Industrial and Environmental Microbiology, Institute of Biological Sciences, Faculty of Biology and Biotechnology, Maria Curie-Skłodowska University, Akademicka 19, 20-033 Lublin, Poland

**Keywords:** synthetic coumarins, human colon cells, viability, cell cycle, antioxidant activity, nitric oxide

## Abstract

In this paper, we will present the synthesis of coumarins bearing a phosphonate group in the C-3 position of the coumarin skeleton and phosphacoumarin derivatives. The compounds were synthesized by Knoevenagel condensation. Notably, the synthetic difficulties in preparing phosphacoumarins have limited previous studies. Our approach allows us to efficiently produce these derivatives, opening the way to investigate their biological properties. The resulting compounds were fully characterized using spectroscopic techniques and high-resolution mass spectrometry. We then evaluated the cytotoxicity of the compounds against human colon cancer HT-29 tumor and CCD 841 CoTr normal colon epithelial cells. We compared these results with coumarin activity to assess the effect of the introduction of the phosphonate group on their cytotoxicity. In addition, we performed cell cycle analysis by flow cytometry and examined the antioxidant activity of the compounds by the DPPH and FRAP methods. Furthermore, we conducted ADME analysis to gain more insight into the pharmacokinetic properties of the tested coumarins. Our study is in line with current trends in the search for new compounds with potential anticancer properties. Although there are numerous reports in the scientific literature on the anticancer activity of coumarin derivatives, the cytotoxicity of synthetic derivatives with a phosphonate group has not been investigated to date.

## 1. Introduction

**Figure 1:**
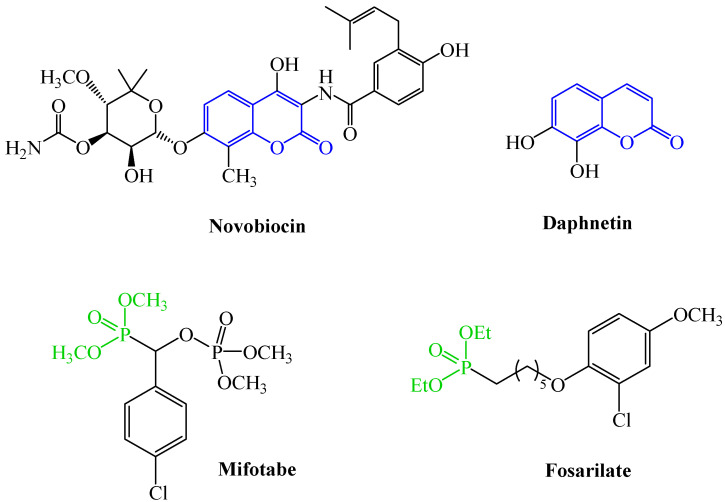
Selected bioactive coumarins and phosphonate derivatives.

Coumarins show great potential as anticancer agents owing to their ability to interact with multiple molecular signaling pathways and inhibit human cancer cell lines [1,2,3,4]. The activities of coumarin derivatives comprise the inhibition of tumor cell proliferation, the modulation of cell-mediated signaling, the suppression of angiogenesis, and apoptosis execution. Coumarins can also repress the expression of some oncogenes and induce oxidative stress through free radical generation, which leads to proapoptotic activities. They also inhibit the growth of tumor cells by blocking cell division at the G0/G1 and G2/M phases [5]. The recent modification of coumarins by adding different pharmacophore prodrugs is a new area of interest. These changes are focused on adding multi-directional activity and improving pharmacokinetic parameters, like lipophilicity and bioavailability. The resulting hybrid compounds are highly promising, as exemplified by novobiocin, which is made up of a noviose sugar, a coumarin backbone, and a benzoyl side chain. Novobiocin’s original use as an antibiotic has come under scrutiny, but the drug has proved useful against tumors that are resistant to PARP inhibitors, especially in patients with BRCA1 or BRCA2 mutations (Figure 1) [6,7].

Phosphonates also have a unique and irreplaceable position in the design and development of drugs and prodrugs [8,9]. They have the ability to interact with various enzymes and affect different metabolic pathways in the body. Phosphonates, such as adenovir and fosarilat, have antiviral effects [10,11], while mifotabe and fostedil are potential cardiovascular drugs [12]. Recently, 2-oxoindolin phosphonate derivatives have been shown to have in vitro anticancer activity against human cancer cell lines, such as MCF-7, IMR-32, SK-MEL-2, MG-63, HT-29, and Hep-G2 [13], while bisphosphonate-based prodrugs are capable of stimulating γδ T cells and inhibiting prenylation in various cancer cell lines [14].

**Figure 2:**
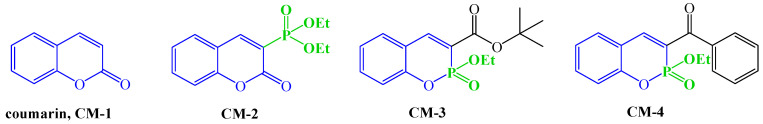
Structures of coumarin and coumarin derivatives for anticancer studies.

Our recent research has focused on the design and synthesis of compounds with potential anticancer activity. Having experience in the chemistry of organophosphorus compounds, we decided to introduce a phosphonate fragment into the known pharmacophore skeleton, coumarin (**CM**-**1**). As the first step of our work, we designed and synthesized three coumarin derivatives: the classical compound **CM**-**2,** with a phosphonate group in the C-3 position, and two phosphacoumarins (**CM**-**3** and **CM**-**4**), with an oxaphosphaheterocyclic group (Figure 2). For all compounds, we undertook in silico analysis of their physicochemical properties and ADME parameters to assess their potential as candidates for further biological studies. Subsequently, we aimed to investigate the impact of the presence of a phosphonate group in the coumarin backbone (**CM**-**2**–**CM**-**4**) on their cytotoxicity against HT-29 tumor and CCD 841 CoTr normal colon epithelial cells.

## 2. Results and Discussion

### 2.1. In Silico Prediction of Physicochemical and ADME Properties

**Figure 3:**
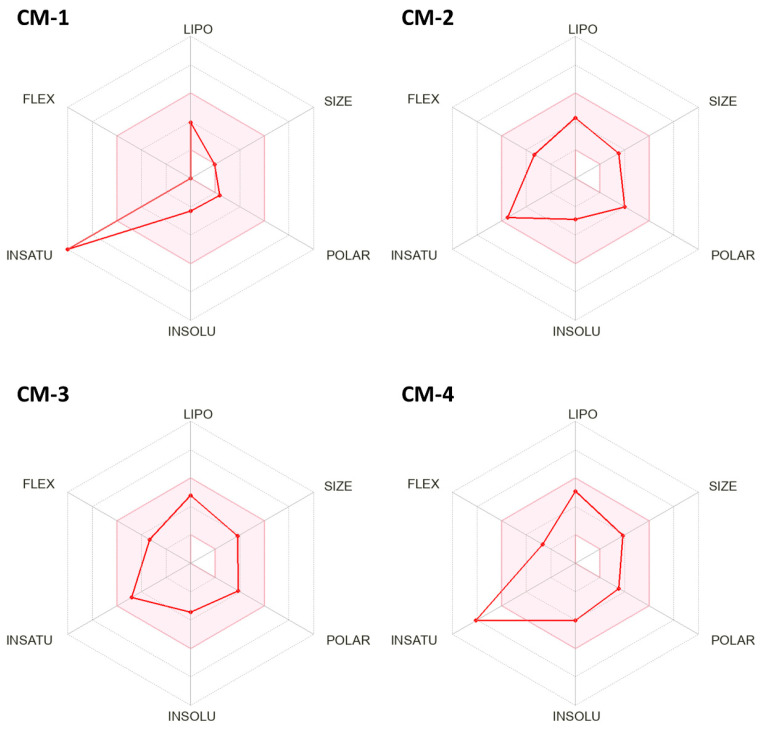
Radar map of bioavailability-related properties. The pink area shows the optimal range for each descriptor (LIPO = −0.7 XLOGP3 5.0; SIZE = 150 g/mol < MW < 500 g/mol, POLAR = 20 Å^2^ < TPSA <130 Å^2^; INSOLU =: −6 < log S (ESOL) < 0; INSATU = fraction of sp^3^ carbons > 0.25; and FLEX = No. of rotatable bonds < 9.

Evaluating physicochemical properties at the early stages of drug discovery, such as hit identification and subsequent optimization, is essential. It helps guide structural modifications toward compounds with favorable pharmacokinetic profiles, which are critically important in the later phases of in vivo testing. To preliminarily assess the drug-likeness, ADME characteristics, and potential toxicity profiles of the synthesized compounds, we performed an in silico analysis. The properties were predicted using the SwissADME platform (http://www.swissadme.ch, accessed on 13 May 2025) [15], a widely recognized computational tool for predicting molecular features relevant to drug development. The calculation results are presented in Table 1 and Table 2 and Figure 3.

According to generally accepted principles in medicinal chemistry [16], the initial structure of the coumarin **CM**-**1** may serve as a representative example of a lead-like compound, with a molecular weight below 300 Da, logP below 3, and no more than three hydrogen bond acceptors, donors, and rotatable bonds (Table 1). Although compounds with this profile often fall short of drug-likeness criteria—as evidenced by violations of the Ghose (molecular weight < 160 Da, fewer than 20 heavy atoms) and Muegge (molecular weight < 200 Da) filters [17] (Table 2)—they can act as a valuable scaffold for the development of larger, more active and selective compounds. This concept was utilized in our research by designing derivatives **CM**-**2**–**CM**-**4**, which fulfil the Lipinski, Ghose, Veber, Egan, and Muegge criteria for drug-likeness and oral bioavailability (Table 2). 

Structural modifications, such as the introduction of a phosphonate at position 3 of the coumarin core (**CM**-**2**), or the transformation of a coumarin into phosphocoumarin with a carbonyl group at position 3 (**CM**-**3** and **CM**-**4**), led to a significant increase in the value of key molecular parameters compared to the initial structure of coumarin **CM**-**1**:

- The topological polar surface area (TPSA) increased from 30.2 Å^2^ to 62.4–75.5 Å^2^, approaching an optimal range that ensures a better balance between lipophilicity and aqueous solubility, and improving the potential for polar interactions with molecular targets.
- The number of hydrogen bond acceptors rose from 2 to 4–5, potentially enabling more extensive hydrogen-bonding.
- Molar refractivity increased from 42.5 to 73.2–85.12, indicating a greater potential for hydrophobic interactions—an important feature that usually constitutes a significant contribution to the Gibbs energy of binding for optimized drug-like molecules [18].
- Average logP values increased from 1.8 to 2.1–3.2, with only a moderate decrease in logS. This balance still supports favorable oral absorption and adequate blood–brain barrier penetration (Table 2).

In terms of the bioavailability, radar plots (Figure 3) reflecting the combined influence of lipophilicity, molecular weight, polarity, solubility, and molecular shape highlight that compounds **CM**-**2** and **CM**-**3** exhibit optimal parameters values conducive to good bioavailability. Moreover, moderate levels of these properties suggest that further structural modification remain possible. All compounds still possess considerable space for molecular size expansion—which typically accompanies structure optimization. Such modifications, affecting polarity, lipophilicity, and solubility—can be introduced, without exceeding the critical thresholds or significantly compromising bioavailability. The addition of appropriate substituents to **CM**-**2** and **CM**-**3** may also improve the fraction of sp^3^-hybridized carbons (INSATU parameter), without surpassing the acceptable limit for rotatable bonds (FLEX < 9). In particular, such optimization is especially desirable for structure **CM**-**4**, given its low sp^3^ carbon fraction.

According to SwissADME predictions, none of the investigated compounds are likely to act as substrates of P-glycoprotein, a key ATP-binding cassette (ABC) transporter involved in multidrug resistance. Furthermore, compounds **CM**-**1**–**CM**-**3** are unlikely to cause inhibition of CYP3A4 and CYP2D6—two major cytochrome P450 isoforms responsible for the metabolism of a broad range of drugs.

Favorable results of ADME analysis and physicochemical properties obtained by the in silico method indicate that compounds **CM**-**2**–**CM**-**4** could be promising candidates for further studies. Therefore, coumarin phosphonate derivatives were synthesized to further evaluate their cytotoxicity against human colon cancer HT-29 tumor and CCD 841 CoTr normal colon epithelial cells. In addition, coumarin (**CM**-**1**) was included as a reference structure to evaluate the effect of the position of the phosphonate group within the coumarin scaffold on the biological activity of the target compounds.

### 2.2. Synthesis of C-3 Phosphonate and Phosphacoumarin Derivatives

**Scheme 1:**
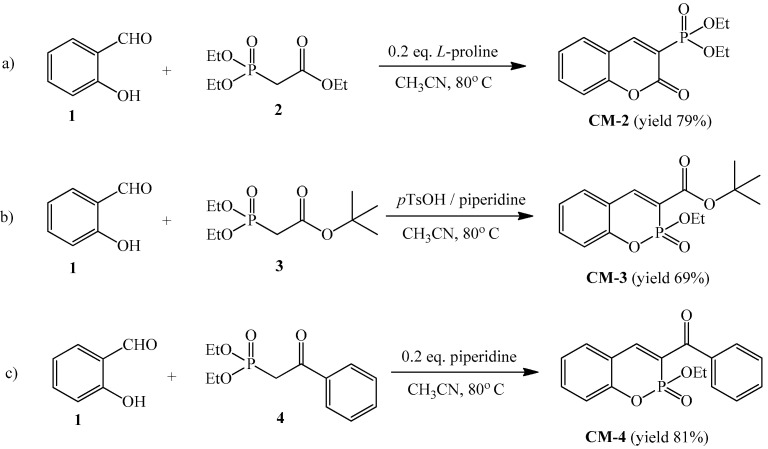
Synthetic route for coumarins **CM**-**2** (**a**), **CM**-**3** (**b**), **CM**-**4** (**c**).

Coumarin **CM**-**1** was obtained from commercial suppliers. Additional compounds were synthesized employing a simple Knoevenagel condensation method [19]. This reaction is commonly used in organic chemistry for the formation of new carbon–carbon bonds. In its classical form, this process occurs at elevated temperatures and requires catalytic amounts of organic bases, including pyrrolidine, piperidine, or pyridine. The synthesis of the compound **CM**-**2** from salicylaldehyde (**1**) and diethoxyphosphoryl-acetic acid ethyl ester (**2**) is known from the literature and proceeds in the presence of piperidine as a catalyst with a reaction yield of 69% [20]. For our synthesis, we applied *L*-proline as a more efficient and safer reactant. By reacting in acetonitrile at 80 °C for 18 h, we achieved the compound **CM**-**2** with a 79% yield (Scheme 1a). In order to prepare phosphacoumarin **CM**-**3**, it was necessary to employ a suitably designed sterically hindered ester **2** (Scheme 1b). Using a catalytic system of *p*-toluenesulphonic acid/piperidine (0.2 eq./0.2 eq.) in acetronitrile for 18 h, it was possible to obtain the **CM**-**3** derivative with a satisfactory yield of 69%. 

For the synthesis of compound **CM**-**4**, the corresponding *β*-ketophosphonate (**4**) was first made according to the procedure described by Chen et al. [21], and then subsequently applied in the condensation reaction with salicylaldehyde (Scheme 1c). We employed piperidine (0.2 eq.) in the reaction, achieving a very good yield of 81% of the desired phosphacoumarin.

Full ^1^H NMR, ^13^C NMR, ^31^P NMR, and mass spectroscopy analyses were used to confirm the structures and purity of all synthesized compounds (Figures S1–S9).

### 2.3. In Vitro Evaluation of Cytotoxicity, Apoptosis Induction, and Antioxidant Activity of ***CM**-**1**–**CM**-**4***

#### 2.3.1. MTT Analysis

**Figure 4:**
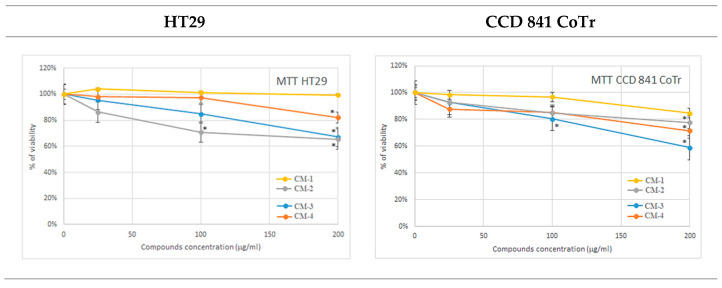
MTT analysis of the metabolic activity of HT29 cells and CCD 841 CoTr cells after 24 h of incubation with tested coumarins. * A *p*-value of ≤0.05 is considered significant.

In the MTT assay we tested the succinyl dehydrogenase activity of cellular mitochondria after cells incubation with coumarins in increasing concentrations (0–200 µg/mL). Coumarin (**CM**-**1**) had no impact on mitochondrial activity of HT29 tumor cells after 24 h of incubation. Compound **CM**-**4**, only at a 200 µg/mL concentration, decreased mitochondrial activity by about 18% compared to the control. The highest action was found for **CM**-**2** and **CM**-**3**, which, at the highest concentration applied (200 µg/mL), reduced activity of the enzyme by 33% and 34.5%, respectively, in comparison to the control (Figure 4).

Normal colonic epithelial cells were more sensitive to the activity of the tested coumarins. Less activity was observed for coumarin **CM**-**1**, where, at the highest concentration used, the mitochondrial activity decreased by about 15.5% compared to the untreated control. The highest action was found for compound **CM**-**2**, which decreased the enzymatic action by 41% compared to the control at a 200 µg/mL concentration of the coumarin (Figure 4). The activity of coumarin derivatives depends, to a large extent, on the substituents, the activity environment, and the type of cells on which they act. Al-Harbi et al. [22] indicated that coumarin derivatives, e.g., amino acid derivatives, may exhibit high potency against human breast cancer cells. An important parameter was the indication that this activity was comparable or higher than the reference drug doxorubicin [22].

#### 2.3.2. NR Uptake Assay 

**Figure 5:**
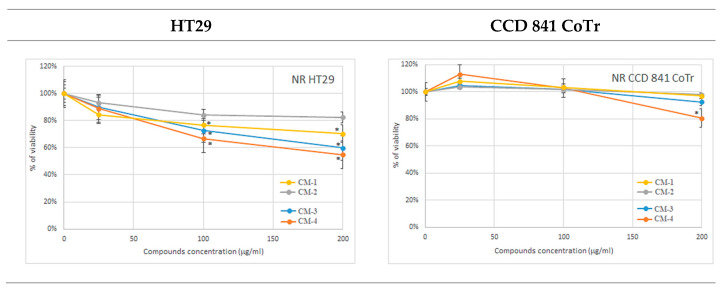
NR uptake assay analysis of the viability of HT29 cells and CCD 841 CoTr cells after 24 h of incubation with tested coumarins.* A *p*-value of ≤0.05 is considered significant.

The lowest cytotoxic activity on tumor HT29 cells was observed for coumarin **CM**-**2**. At the highest concentration applied, the viability decreased by about 17.5% compared to the control. The same concentration of coumarin **CM**-**4** decreased the viability of HT29 cells by about 45% in comparison to the untreated control (Figure 5).

The tested coumarins had no significant cytotoxic impact on normal human colon epithelial cells, with the exception of the highest concentration of compound **CM**-**1**. The decrease in viability was 19.5% compared to the control (Figure 5).

Coumarins with additional substituents may exhibit high antitumor activity in relation to the parent coumarin. Rahana et al. studied coumarin–chalcone derivatives and showed high activity in reducing the viability of human breast cancer cells in relation to the parent compound [23]. Similarly, this tumor may be a good target for 3-phenyl coumarin derivatives, which significantly reduce the viability of this tumor cell line. Moreover, Jevtić et al. showed that the coumarin–palladium complex may have strong cytotoxic activity directed at tumor cells of different origin, including those derived from human colon cancer [24]. Hydrazide–hydrazone derivatives also exhibit cytotoxic and proapoptotic activity on liver cancer cells [25].

#### 2.3.3. Cell Cycle Analysis

**Figure 6:**
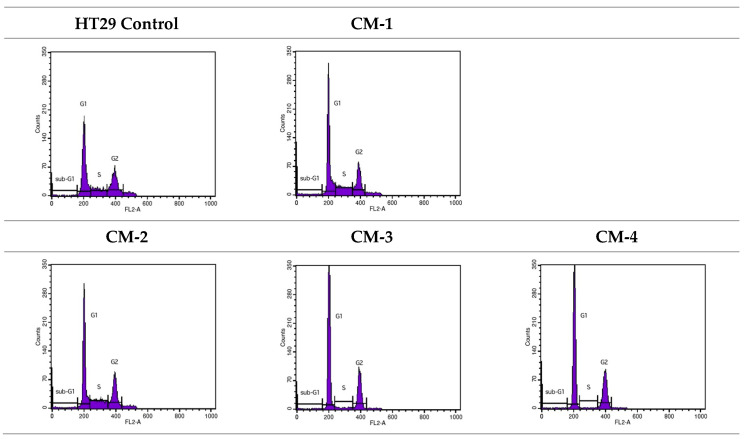
Flow cytometry analysis of changes in the cell cycle phases in HT29 human colon tumor cells after 24 h of incubation with the tested coumarins at a 200 μg/mL concentration. Only representative DNA histograms are presented.

Coumarins **CM**-**1**, **CM**-**2**, and **CM**-**4** increased the number of HT29 cancer cells in the sub-G1 phase. This suggests that the tested substances stimulated the apoptosis process. Cells were blocked in the G1 phase after incubation with coumarins **CM**-**3** and **CM**-**4**. Therefore, a significant decrease in the number of cells in the S phase was observed in the same samples compared to the untreated control (Figure 6 and Table S1).

**Figure 7:**
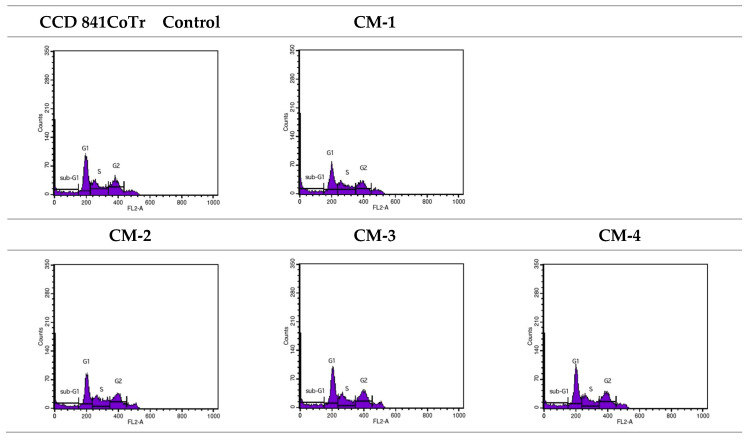
Flow cytometry analysis of changes in the cell cycle phases in CCD 841 CoTr human normal colon epithelial cells after 24 h of incubation with the tested coumarins at the 200 μg/mL concentration. Only representative DNA histograms are presented.

In the case of normal intestinal epithelial cells, an increase in the number of cells in the sub-G1 phase was also observed after their incubation with coumarins **CM**-**1**, **CM**-**2**, and **CM**-**4**. In the case of coumarin **CM**-**1**, an approximately 4% decrease in the cell’s number in the G1 phase was also demonstrated compared to the control. Coumarins **CM**-**3** and **CM**-**4** also blocked the transition of cells from the S phase to G2. However, this was not as strong a blockade as in the case of cancer cells. There were no significant changes in the number of both normal and neoplastic cells in the G2 phase compared to the control (Figure 7 and Table S1).

Coumarin derivatives affect the distribution of cells in different phases of the cell cycle. The main changes concern the G1 and S phases. The results of our studies confirm the observations of Khosravifar et al., indicating that selected synthetic coumarin derivatives most significantly affect these phases of the cycle [26]. A similar effect was observed for esculetin, a natural coumarin derivative, on breast cancer cells [27]; for 4-flourophenylacetamide-acetyl coumarin on human lung cancer cells [28]; and for thiazolylpyrazolyl coumarin derivatives on both types of cancer [29]. The basis for such activities could be the change in the ratio of P53 protein to Bax/Bcl-2 or VEGFR-2 kinase in the cells. Moreover, the studied coumarin derivatives could significantly affect the activation of selected molecular pathways, such as AKT, and change the local concentrations of selected molecules (ROS, NO, or p21 protein), affecting the proliferative capacity of cells. Additionally, coumarin derivatives influence the activation of certain cyclins and cyclin-dependent kinases, which are important for the cell to move through different stages of interphase. In the case of G1 and S phases, these would be CDK2, CDK4, and CDK6, and cyclins D, E, or G1. Their regulation, therefore, significantly affects cell proliferation and, in the context of cancer, its growth.

#### 2.3.4. Quantitative Analysis of Cell Death Types

**Figure 8:**
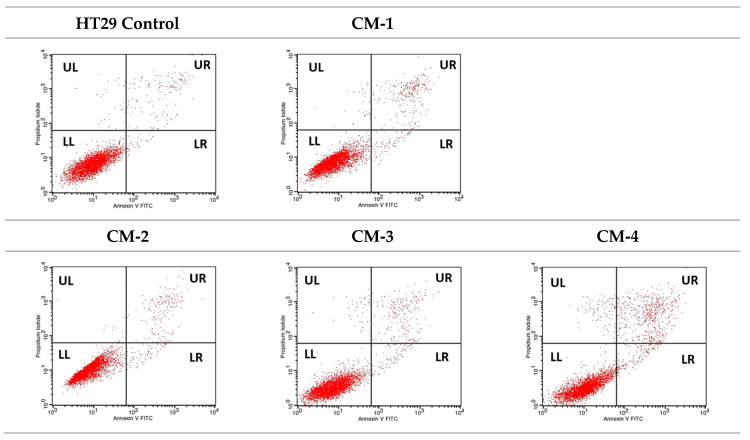
Apoptosis/necrosis level after the incubation of HT29 tumor cells with tested coumarins at the 200 μg/mL concentration. Flow cytometry analysis after cell staining with annexin V-FITC and propidium iodide. Only representative dot plots are presented. An−/PI+ necrotic cells are located the UL Quad, An+/PI+ late apoptotic cells in the UR Quad, An−/PI− viable cells in the LL Quad, and An+/PI− early apoptotic cells in the LR Quad.

The most pronounced effect on changes in the viability of HT29 cancer cells was exerted by coumarin **CM**-**4**. It caused a decrease in cell viability by about 11.6% compared to the untreated control. These cells died mainly by late apoptosis (Figure 8 and Table S2).

**Figure 9:**
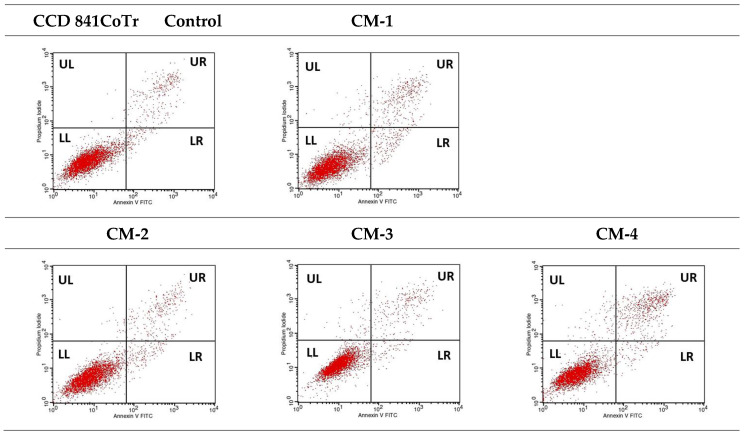
Apoptosis/necrosis levels after the incubation of CCD 841CoTr cells with tested coumarins at a 200 μg/mL concentration. Flow cytometry analysis after cell staining with annexin V-FITC and propidium iodide. Only representative dot plots are presented. An−/PI+ necrotic cells are located in the UL Quad, An+/PI+ late apoptotic cells in the UR Quad, An−/PI− viable cells in the LL Quad, and An+/PI− early apoptotic cells in the LR Quad.

Similarly, coumarin **CM**-**4** reduced the viability of normal intestinal epithelial cells by about 8.6% compared to the control. The decrease in viability was also associated with an increase in the number of cells in late apoptosis. Statistically significant cytotoxic activity was also noted for coumarin **CM**-**1**, which, however, caused a loss of viability, mainly through necrosis compared to the control (Figure 9 and Table S3).

Apoptosis is an interesting defense mechanism against cancer cell proliferation and brings tangible benefits to tissues compared to necrotic dying. In our studies, we mainly indicated late apoptosis as a way of eliminating cancer cells by the studied coumarin derivatives. Studies by other authors have also indicated that various coumarin derivatives reduce the viability of cancer cells by apoptosis [30,31,32]. The explanation for these observations is the significant effect of the studied derivatives on molecules and molecular pathways in the studied cells. The action of coumarins on proteolytic cascade activation and consequently caspase-3 activity and poly ADP-ribose polymerase (PARP) expression is indicated. In addition, there may be a loss of the antiapoptotic protein level and a simultaneous increase in the amount of proapoptotic proteins, such as Bak. Moreover, coumarins derivatives may have a regulatory effect on molecular pathways in the cell, mainly on the PI3K/Akt/mTOR signaling pathway, which, in consequence, will affect cell proliferation and their apoptotic death [32].

#### 2.3.5. Nitric Oxide Level Analysis—Griess Method

**Figure 10:**
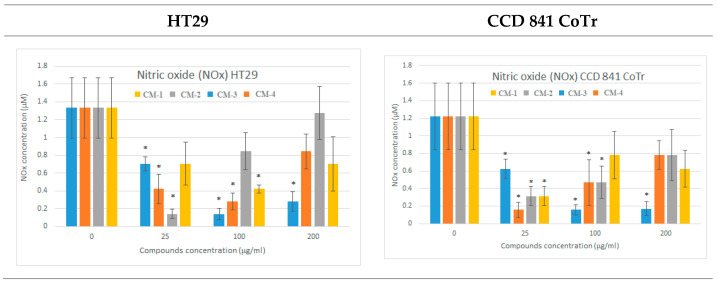
Nitric oxide (NOx) level analysis produced by HT29 cells and CCD 841CoTr cells after 24 h of incubation with the tested coumarins, using the Griess reaction.* A *p*-value of ≤0.05 is considered significant.

The tested coumarins led to a decrease in the amount of nitric oxide released by both cancer and normal cells. The level of changes in both cell types was also comparable. No clear relationship was found between the concentration of coumarins used and the level of NOx reduction in post-culture fluids. Specifically, the following was observed:

In cancer HT29 cell cultures, coumarin **CM**-**3** reduced the amount of NOx with increasing concentration, coumarin **CM**-**4** increased the amount of detected NOx with the use of a higher concentration of the compound, coumarin **CM**-**2** stimulated the release of higher amounts of NOx with increasing concentration, and coumarin **CM**-**1** did not show a trend in NOx level changes with an increasing concentration of the tested compound (Figure 10).

In the culture of normal cells, coumarin **CM**-**3** reduced the amount of NOx with increasing compound concentrations, coumarins **CM**-**4** and **CM**-**2** increased the amount of detected NOx with increasing compound concentrations, while coumarin **CM**-**1**, similarly to HT29 cells, did not show a clear trend in changes in the NOx level depending on the compound concentration (Figure 10).

The inhibition of NO release by cells exposed to coumarin derivatives was confirmed by El-Gamal et al. They demonstrated a limiting effect of tetracyclic-fused coumarin sulfonate on the production of this molecule by macrophage cells [33]. In our studies, the inhibition concerned cancer cells as well as normal cells. This may be related to the effect of the tested derivatives on nitric oxide synthases, specifically the constitutively expressed ones in our case, but it is possible that coumarins also regulate the activity of the inducible form of NOS. We believe that such an effect could exist both at the post-translational level and occur at the level of mRNA for specific synthases. Moreover, coumarins may have general antioxidant and anti-inflammatory characteristic effects associated with the level of NO. This regulation is based on their effect on the arachidonic acid pathway, changes in cyclooxygenase (COX-2) activity, and the regulation of potential inflammation [34].

#### 2.3.6. DPPH Antioxidant Activity Analysis

All tested coumarins showed activity reducing the free DPPH radical. This activity was concentration-dependent. The strongest DPPH radical scavenging effect was observed for coumarin **CM**-**2**. At a concentration of 200 µg/mL, the reducing activity of this coumarin was equivalent to Trolox activity at a concentration of 25.3 µg/mL. The weakest activity was shown for coumarin **CM**-**1**, which, at a concentration of 200 µg/mL, showed a reducing activity equivalent to Trolox action at a concentration of 3.2 µg/mL (Table S4).

#### 2.3.7. FRAP Analysis

All tested coumarins showed activity reducing the Fe^3+^ ion. This activity was similar to that observed in the DPPH method and was dependent on the concentration of the tested coumarin. The highest reducing activity was observed for coumarin **CM**-**2**, which, at a concentration of 200 µg/mL, showed a reducing activity equivalent to 9 µg/mL of ascorbic acid. The lowest activity was noted for coumarin **CM**-**3**. At the highest applied concentration (200 µg/mL), it reduced the Fe3+ ion in a manner equivalent to ascorbic acid at a concentration of 2 µg/mL (Table S5).

Our studies confirm the results of other authors, indicating the antioxidant activity of selected coumarin derivatives [34,35,36]. The diverse antioxidant activity of coumarins is due to the complexity of their action mechanisms. An important role is also played by the types of substituents that can react with DPPH or reduce the iron ion. The mechanisms of intracellular antioxidant action of coumarin derivatives are based mainly on the regulation of the transcription factor NF-κB and, consequently, all downstream processes. In addition, the JNK1/2 and p38 MAPK signaling pathways are subject to the influence of coumarin derivatives. This certainly has an impact on many processes in the cell, including the expression of pro- and anti-inflammatory activity, as well as redox effects based, among others, on the activity of COX-2 or PGE2.

#### 2.3.8. MGG Staining

**Figure 11:**
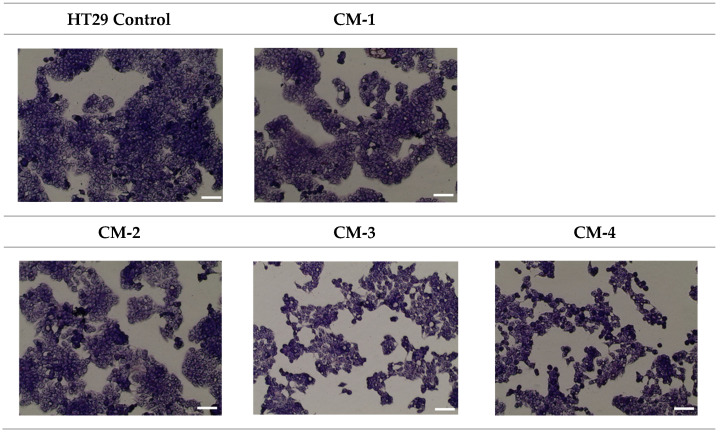

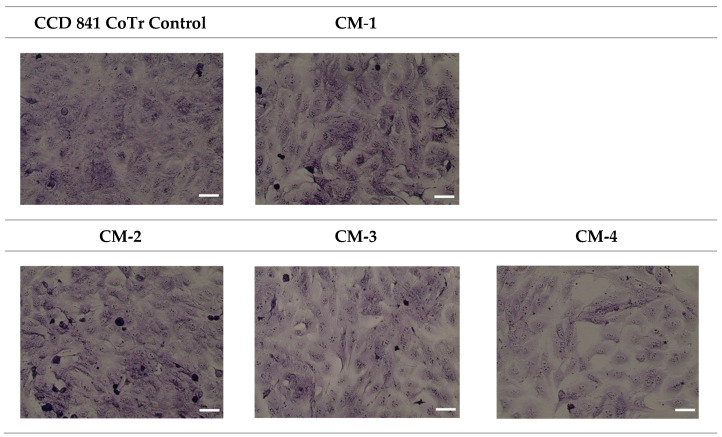
May–Grünwald–Giensa (MGG) staining of HT-29 tumor and CCD 841 CoTr normal colon epithelial cells. Cells were incubated for 24 h with tested coumarins at a 200 µg/mL concentration. Magnification 100×. Bar = 50 µm.

The staining of cells using the MGG method confirmed the observations of cell viability made using spectrophotometric methods. Cancer cells after incubation with coumarins **CM**-**3** and **CM**-**4** showed reduced culture density, reduced cell adhesion to the substrate, and a reduced degree of culture compactness compared to the control. Coumarins **CM**-**1** and **CM**-**2** only caused a decrease in the number of cells compared to the untreated control. Normal cells did not show significant changes in morphology compared to the control after incubation with the tested compounds. Only a decrease in cell culture density was observed, especially after incubation with coumarin **CM**-**4** (Figure 11).

## 3. Materials and Methods

### 3.1. Chemistry

General: All commercially available chemicals and solvents obtained were of high quality and employed without further purification. NMR spectra were recorded using a Bruker AV500 (^1^H 500 MHz, ^13^C 126 MHz, ^31^P 202 MHz) spectrometer (Billerica, MA, USA). All spectra were obtained in CDCl_3_ solutions, and the chemical shifts (δ) were expressed in ppm using an internal reference to TMS. Coupling constants (*J*) were given in Hz. The abbreviations of the signal patterns were as follows: s, singlet; d, doublet; t, triplet; q, quartet; m, multiplet; b, broad. Melting points were determined on a Buchi 510 apparatus. Thin-layer chromatography (TLC) was performed on silica gel (Kieselgel 60, F254 on aluminum sheets, Merck, Darmstadt, Germany) using UV light (254 nm). HPLC–HRMS was performed on a Shimazu LCMS-8030 LCMS System(Shimadzu Europa, Duisburg, Germany) using a reverse-phase stationary phase with water/MeCN (65:35) as an eluent, electrospray ionization (ESI), and an IT-TOF detector (Shimadzu Europa, Duisburg, Germany). 

#### 3.1.1. Synthesis of Diethyl (2-Oxo-2H-chromen-3-yl)phosphonate (**CM**-**2**)

Salicylaldehyde (**1**, 1.0 g, 8.18 mmol), diethoxyphosphoryl-acetic acid ethyl ester (**2**, 2.20 g, 9.81 mmol, 1.2 eq.), *L*-proline (0.180 g, 0.2 eq.), and acetonitrile (25 ml) were placed in a Schlenk tube. The mixture was stirred at 80 °C for 18 h. Subsequently, the volume of the solvent was evaporated. The residue was purified by column chromatography on silica gel (DCM/MeOH 50:1) to obtain **CM**-**2**.

White solid, M.p. of 61–63 °C, Rf of 0.6 (DCM/MeOH 15:1), yield of 79%. ^1^H NMR (500 MHz, Chloroform-*d*) δ 8.51 (dd, *J* = 17.2, 0.7 Hz, 1H), 7.67–7.57 (m, 2H), 7.36–7.30 (m, 2H), 4.35–4.17 (m, 4H), 1.36 (td, *J* = 7.1, 0.7 Hz, 6H). ^13^C NMR (126 MHz, Chloroform-*d*) δ 158.21 (d, *J* = 14.5 Hz), 155.25, 153.50 (d, *J* = 6.6 Hz), 134.26, 129.38, 124.91, 117.92 (d, *J* = 14.2 Hz), 117.72 (d, *J* = 196.8 Hz), 116.86, 63.44 (d, *J* = 5.9 Hz), 16.36 (d, *J* = 6.3 Hz). ^31^P NMR (202 MHz, Chloroform-*d*) δ 10.91. The NMR data are consistent with the values reported for **CM**-**2** in the literature [20].

#### 3.1.2. Synthesis of *t*-Butyl (2-Ethoxy-2-oxo-2H-1,2-benzoxaphosphorine)-3-carboxylate (**CM**-**3**)

Salicylaldehyde (**1**, 1.0 g, 8.18 mmol), diethoxyphosphoryl-acetic acid *t*-butyl ester (**3**, 2.47 g, 9.81 mmol, 1.2 eq.), *p*TsOH monohydrate (0.320 g, 0.2 eq.), piperidine (0.140 g, 0.2 eq.), and acetonitrile (25 ml) were placed in a Schlenk tube. The mixture was stirred at 80°C for 18 h. Subsequently, the volume of the solvent was evaporated. The residue was purified by column chromatography on silica gel (DCM/MeOH 100:1) to obtain **CM**-**3**.

Colorless oil, Rf of 0.4 (DCM/MeOH 25:1), yield of 69%. ^1^H NMR (500 MHz, Chloroform-d) δ 8.14 (d, *J* = 37.4 Hz, 1H), 7.48–7.40 (m, 2H), 7.21–7.14 (m, 2H), 4.44–4.26 (m, 2H), 1.58 (d, *J* = 1.0 Hz, 9H), 1.40 (t, *J* = 7.1 Hz, 3H). ^13^C NMR (126 MHz, Chloroform-*d*) δ 162.74 (d, *J* = 12.6 Hz), 152.62 (d, *J* = 8.9 Hz), 149.78 (d, *J* = 4.0 Hz), 133.39, 131.49 (d, *J* = 1.7 Hz), 124.06, 120.58, 119.62 (d, *J* = 15.9 Hz), 119.18, 118.71 (d, *J* = 7.5 Hz), 83.06, 64.61 (d, *J* = 6.4 Hz), 28.14, 16.47 (d, *J* = 6.5 Hz). ^31^P NMR (202 MHz, Chloroform-*d*) δ 5.57. LCMS: [M+H]^+^ m/z calcd for C_15_H_20_O_5_P^+^: 311.1048, found: 311.1047.

#### 3.1.3. Synthesis of 3-Benzoyl-(2-ethoxy-2-oxo-2H-1,2-benzoxaphosphorine) (**CM**-**4**)

Salicylaldehyde (**1**, 1.0 g, 8.18 mmol), *β*-ketophosphonate (**4**, 2.50 g, 9.81 mmol, 1.2 eq.), piperidine (0.140 g, 0.2 eq.), and acetonitrile (25 ml) were placed in a Schlenk tube. The mixture was stirred at 80 °C for 18 h. Subsequently, the volume of the solvent was evaporated. The residue was purified by column chromatography on silica gel (DCM/MeOH 100:1) to obtain **CM**-**4**.

Colorless oil, Rf of 0.5 (DCM/MeOH 25:1), yield of 81%. ^1^H NMR (500 MHz, Chloroform-d) δ 7.85–7.80 (m, 2H), 7.76 (d, *J* = 38.9 Hz, 1H), 7.65–7.61 (m, 1H), 7.54–7.49 (m, 3H), 7.38 (dd, *J* = 7.7, 1.7 Hz, 1H), 7.25–7.20 (m, 2H), 4.54–4.40 (m, 2H), 1.40 (td, *J* = 7.1, 0.6 Hz, 3H). ^13^C NMR (126 MHz, Chloroform-*d*) δ 193.08 (d, *J* = 7.5 Hz), 152.40 (d, *J* = 8.4 Hz), 149.50 (d, *J* = 3.1 Hz), 136.55 (d, *J* = 7.0 Hz), 133.76, 132.98, 131.56 (d, *J* = 1.7 Hz), 129.48, 128.70, 126.88 (d, *J* = 168.6 Hz), 124.32, 119.87 (d, *J* = 17.2 Hz), 119.15 (d, *J* = 7.2 Hz), 65.07 (d, *J* = 6.0 Hz), 16.41 (d, *J* = 6.5 Hz). ^31^P NMR (202 MHz, Chloroform-*d*) δ 6.46. LCMS: [2M+Na]^+^ m/z calcd for C_34_H_30_O_8_P_2_^+^: 650.1291, found: 650.1308.

### 3.2. Biology

#### 3.2.1. Cell Cultures

The HT29 cell line (ATCC no. HTB-38), derived from human colon adenocarcinoma, was utilized in the investigation. Cells were grown in RPMI 1640 medium supplemented with 10% fetal calf serum (FCS) (GibcoTM, Paisley, UK) and antibiotics (100 U/mL penicillin and 100 μg/mL streptomycin) (Sigma, St. Louis, MO, USA). The culture was conducted under conventional conditions, specifically at 37 °C in a humidified environment with 5% CO_2_. 

The CCD 841 CoTr cell line (ATCC no. CRL-1807), derived from human normal colon epithelium, was cultured in a 1:1 mixture of RPMI 1640 and DMEM (Sigma, St. Louis, MO, USA), supplemented with 10% fetal calf serum and antibiotics. The culture was maintained at 34 °C in a 5% CO_2_ and 95% air environment.

#### 3.2.2. MTT Assay

Cells were inoculated at a density of 1 × 10^5^ cells/mL in a 96-well plate and incubated for 24 h. Subsequently, coumarins (100 μL) at doses of 0, 25, 100, and 200 μg/mL were introduced into the wells containing the growth medium (100 μL). The incubation continued for an additional 24 h. Subsequently, an MTT solution (5 mg/mL, 25 μL/well) was introduced to each well. The incubation was extended for an additional 3 h. Subsequent to the incubation, 100 μL of 10% sodium dodecyl sulfate (SDS) in 0.01 M HCl was introduced to solubilize the purple formazan crystals. Solubilization was conducted overnight. Ultimately, spectrophotometric analysis was conducted at 570 nm utilizing an EL800 Universal Microplate Reader (BioTek Instruments, Winooski, VT, USA).

#### 3.2.3. Neutral Red Uptake Assay

Cells were inoculated at a density of 1 × 10^5^ cells/mL on a 96-well plate, followed by a 24-h incubation period. Subsequently, coumarins (100 μL) at concentrations of 0, 25, 100, and 200 μg/mL were introduced into the wells containing the culture medium (100 μL). The incubation continued for an additional 24 h. Subsequently, the culture medium was discarded, and a 0.4% Neutral Red solution in the culture medium was introduced to each well. The incubation persisted for 3 h. Subsequent to incubation, the solution containing the NR dye was eliminated, and 200 μL of 4% paraformaldehyde in 1% CaCl_2_ was introduced for cell fixation and incubated for 3 min. Intracellular dye deposits were solubilized with 1% acetic acid in a 50% ethanol solution (100 μL). The plates were gently agitated for 20 min at room temperature to dissolve and extract the dye from the cells. Ultimately, spectrophotometric analysis was conducted at 540 nm utilizing an EL800 Universal Microplate Reader (BioTek Instruments, Winooski, VT, USA). 

#### 3.2.4. Cytometric Assessment of the Cell Cycle

The effect of coumarins at a concentration of 200 μg/mL on the cell cycle phases in normal and cancer cells was evaluated after 24 h of incubation. The cells were harvested following 24 h of incubation with the experimental chemicals and subjected to centrifugation at 3000 rpm for 5 min. The cell pellet was washed and resuspended in PBS devoid of Ca^2+^ and Mg^2+^ ions, then centrifuged at 3000 rpm for 5 min. The cell pellet was resuspended and fixed in 1 mL of 70% ethanol. Untreated cells functioned as a control group. The cellular samples were preserved at −20 °C for one week. Subsequently, the cells underwent the PI staining technique with the PI/RNase Staining Buffer from BD PharmingenTM (BD Biosciences, San Jose, CA, USA). In summary, 1 mL of PBS devoid of Ca^2+^ and Mg^2+^ ions was combined with 1 mL of ethanol and incubated at ambient temperature with gentle agitation for 5 min. The cells were subsequently centrifuged at 3000 rpm for 5 min and resuspended in PBS devoid of Ca^2+^ and Mg^2+^ ions, with gentle stirring for 5 min at room temperature. The cells underwent centrifugation at 3000 rpm for 5 min, after which the cell pellet was resuspended in 500 μL of PI/RNase Staining Buffer. The incubation was conducted for 15 min at ambient temperature. The intensity of the PI fluorescence was quantified with FACS Calibur (BD PharmingenTM, San Jose, CA, USA). The acquired data were analyzed with Cell Quest Pro Version 6.0 for the Macintosh operating system (BD PharmingenTM, San Jose, CA, USA). The percentage of cells in the relevant phases of the cycle was determined based on all assessed gated cells. A total of 10,000 events were quantified each sample.

#### 3.2.5. Quantitative Analysis of Cell Death Types Using Flow Cytometry

The effects of coumarins at a concentration of 200 μg/mL on the types of normal and tumor cell mortality after 24 h of incubation were evaluated. The annexin V-fluorescein isothiocyanate (FITC)/propidium iodide (PI) apoptosis kit (BD Biosciences, BD PharmingenTM, San Jose, CA, USA) was employed for the quantitative assessment of the distribution of cell death types. To remove adherent cells, a solution of 10 mM EDTA in PBS devoid of Ca^2+^ and Mg^2+^ ions was employed. For the analyses, both adherent and suspended cells were harvested after 24 h of incubation with the evaluated coumarins. The cells were gently centrifuged (1000 rpm for 5 min), the supernatant was discarded, and the cell pellet was resuspended in warm PBS devoid of Ca^2+^ and Mg^2+^ ions and incubated for 5 min at room temperature. The samples were subjected to centrifugation once more, and the cells were resuspended in 100 μL of 1X Annexin V Binding Buffer, supplemented with 5 μL of FITC Annexin V (5 mM) and 5 μL of propidium iodide (PI) (5 mM). The preparation was gently vortexed and incubated at ambient temperature for 15 min in the absence of light. The cells underwent cytometric analysis. Prior to administering the cells into the cytometer, 400 μL of 1X Binding Buffer was incorporated. The cells were examined utilizing a flow cytometer (BD FACSCalibur) with CellQuest Pro Version 6.0 software within one hour of staining completion.

#### 3.2.6. Measurement of Nitric Oxide (NOx)

The spectrophotometric Griess method was utilized for the determination of NOx levels. Nitrate, a stable end product of nitric oxide, was analyzed in the culture supernatants. Following 24 h of cell incubation with the examined coumarins, the culture media was harvested for examination. In summary, 100 μL of the supernatant was dispensed into 96-well flat-bottom plates in triplicate and incubated with 100 μL of Griess reagent (1% sulphanilamide/0.1% N-(1-naphthyl)ethylenediamine dihydrochloride) (Sigma, St. Louis, MO, USA) in 3% H_3_PO_4_ (POCH Gliwice, Poland) at ambient temperature for 10 min. The optical density was assessed at 550 nm utilizing a microplate reader (BioTek Instruments, Winooski, VT, USA). A standard curve was established utilizing 0.5–25 μM sodium nitrite (NaNO_2_) for the calibration and quantitative assessment of NOx levels in the analyzed samples.

#### 3.2.7. DPPH Free Radical Scavenging Assay

The free radical scavenging activity of the evaluated coumarins was assessed using the 1.1-diphenyl-2-picrylhydrazyl (DPPH) assay, which analyzed the capacity of antioxidants to convert the stable dark violet radical DPPH (Sigma, St. Louis, MO, USA) into the yellow diphenyl-picrylhydrazine. In summary, 100 μL of a DPPH solution (0.2 mg/mL in ethanol) was combined with 100 μL of coumarin concentrations (0, 25, 100, and 200 μg/mL) and standards. Trolox (Sigma, St. Louis, MO, USA) was utilized as a reference for free radical scavenging activity at escalating concentrations (1–50 μg/mL). Following a 20-min incubation at ambient temperature, the absorbance was assessed at 515 nm utilizing an EL800 Universal Microplate Reader (BioTek Instruments, Winooski, VT, USA). A reduced absorbance signifies enhanced free radical scavenging ability of the evaluated substances. The efficacy of the coumarins was assessed by comparing their absorbance to that of a blank solution (reagents devoid of coumarins) and a standard. The ability to scavenge the DPPH radical was determined using the subsequent formula: 

DPPH scavenging effect (%) = [(X_control_ − X_coumarin_)/X_control_] × 100, where X_control_ represents the absorbance of the control, and X_coumarin_ denotes the absorbance in the presence of the chosen coumarin.

#### 3.2.8. Ferric-Reducing Antioxidant Power Assay (FRAP)

The quantities of coumarins were dissolved in Milli-Q water and combined with an equivalent volume of 0.2 M sodium phosphate buffer (pH 6.6) and 1% potassium ferricyanide. The mixture was incubated for 30 min at 37 °C. Subsequently, 10% trichloroacetic acid (w/v) was incorporated, and the mixture was centrifuged at 1000 rpm for 5 min. One milliliter of the upper layer was combined with an equal volume of Milli-Q water and 0.1% ferric chloride. The absorbance was measured at 700 nm utilizing an EL800 Universal Microplate Reader (BioTek Instruments, Winooski, VT, USA). Ascorbic acid (0–150 μg/mL) served as a positive control.

#### 3.2.9. May–Grünwald–Giemsa (MGG) Staining

The cells were incubated in 24-well plates with 1 mL of culture media enriched with the evaluated coumarins. Following 24 h of incubation at 37 °C in a humidified atmosphere of 5% CO_2_ and 95% air, the media was removed, the cell cultures were washed with culture medium, and subsequently stained with May–Grünwald (MG) stain for 5 min at room temperature. The MG dye was subsequently diluted with an equivalent volume of water, and the staining process was continued for an additional 5 min. The MG was eliminated, and Giemsa reagent (diluted 1:20 in water) was introduced, followed by a 15-min incubation at room temperature. Subsequently, the cells were washed thrice with water, desiccated, and examined microscopically (Olympus, BX51; Olympus, Tokyo, Japan).

### 3.3. Statistical Evaluation

All experiments were conducted in triplicate, and the data are reported as means ± SD (standard deviation). Data analysis was conducted using one-way ANOVA accompanied by Dunnett’s post hoc test. Differences with *p* ≤ 0.05 were deemed significant.

## 4. Conclusions

This study presents the synthesis of coumarin derivatives with a phosphonate group in the C-3 position and two phosphacoumarins with an oxaphosphaheterocyclic group (**CM**-**3** and **CM**-**4**). The synthesis process involved the classical Knoevenagel condensation reaction, which afforded compounds with very good yields. Swiss-ADME analysis indicated that the **CM**-**2**–**CM**-**4** compounds have the optimal physicochemical parameters required for drug-like molecules. Importantly, there remains considerable room for further structure expansion during their potential optimization without compromising their bioavailability. We demonstrated a differentiated effect of phosphonate coumarin derivatives toward HT-29 colon cancer cells and CCD 841 CoTr human normal colon epithelium cells. The coumarins showed cytotoxic activity, affected the cell cycle, and thus regulated the apoptosis and proliferation of the tested cells. Antioxidant properties of the tested derivatives were also observed based on direct analyses and on changes in the level of nitric oxide.

In summary, the findings indicate that the introduction of a phosphonate moiety significantly affects the physicochemical and biological properties of coumarin derivatives. Compound **CM**-**2** demonstrated the best profile overall, combining favorable in silico parameters with moderate biological activity. Further refinement of the coumarin structure will be necessary to obtain compounds with higher efficacy and selectivity. This is the next step that our team is currently advancing.

## Figures and Tables

**Table 1:** Predicted physicochemical properties of compounds **CM**-**1**–**CM**-**4**.

CM	M.W.	Fsp^3^	NRB	HBA	MR	TPSA	Av. LogP	XLogP3	ESOL Log S	Ali Log S	Silicos-IT LogSw
**CM**-**1**	146.14	0.00	0	2	42.48	30.21	1.82	1.39	−2.29	−1.63	−3.59
**CM**-**2**	282.23	0.31	5	5	73.24	75.55	2.13	1.96	−2.88	−3.17	−4.69
**CM**-**3**	310.28	0.40	5	5	80.82	71.64	2.84	2.84	−3.43	−4.00	−3.87
**CM**-**4**	314.27	0.12	4	4	85.18	62.41	3.19	3.34	−4.03	−4.33	−5.44

**M.W.**—molecular weight; **Fsp^3^**—fraction of sp^3^ carbons; **NRB**—number of rotatable bonds; **HBA**—number of H-bond acceptors; **MR**—molar refractivity; **Av. LogP**—average logP value of different algorithms (iLogP, XLogP3, WLogP, MLOGP, Silicos-IT Log P); **XLogP3**—logP value predicted with XlogP3 algorithm; **ESOL Log S**, **Ali Log S**, **Silicos-IT LogSw**—logS values predicted with corresponding algorithms.

**Table 2:** Selected prediction of ADME properties and drug-likeness filters.

		CM-1	CM-2	CM-3	CM-4
**Pharmacokinetics predictions**	GIT Absorption	High	High	High	High
	BBB permeation	Yes	Yes	Yes	Yes
	P-glycoprotein substrate	No	No	No	No
**CYP450** **inhibition**	CYP3A4	No	No	No	Yes
	CYP2D6	No	No	No	No
	Cyp1A2	Yes	Yes	Yes	Yes
	Cyp2C19	No	Yes	Yes	Yes
	Cyp 2C9	No	No	Yes	Yes
**Drug-likeness** **filter violations**	Lipiniski	0	0	0	0
	Ghose	2	0	0	0
	Veber	0	0	0	0
	Egan	0	0	0	0
	Muegge	1	0	0	0

## Data Availability

The raw data supporting the conclusions of this article will be made available by the authors on request.

## Supplementary Materials

The following supporting information can be downloaded at https://www.mdpi.com/article/10.3390/molecules30132846/s1: Biological data summary and analysis (Tables S1–S5) and NMR spectra (1H, 13C, and 31P) of compounds CM-2, CM-3, and CM-4 (Figures S1–S9), and high-resolution mass spectra (HRMS) for **CM**-**2** and **CM**-**4**.
