# Supplementary material for: Modulation of Human Colon Cell Activity by Synthetic Coumarin Derivatives Bearing a Phosphonate Group
Source: Molecules. 2025 Jul 3;30(13):2846. doi: 10.3390/molecules30132846 (PMC12251547; doi:10.3390/molecules30132846)
Supplement: Supplementary file 1 [file molecules-30-02846-s001.zip › molecules-3710155-supplementary.pdf]

Supplementary Materials

Modulation of Human Colon Cells Activity by Synthetic Coumarin Derivatives Bearing a Phosphonate Group

Katarzyna Szwaczko [1], Roman Paduch [2,3,*], Kamil Dziuba [1], Krzysztof Szafrański [4] and Adrian Wiater [5,*]

[1] Department of Organic Chemistry and Crystallochemistry, Institute of Chemical Sciences, Faculty of Chemistry, Marie Curie-Skłodowska University, Gliniana 33, 20-614 Lublin, Polan; katarzyna.szwaczko@mail.umcs.pl (K.Szwaczko); kamil.dziuba@mail.umcs.pl (K.D.)
[2] Department of Virology and Immunology, Institute of Biological Sciences, Faculty of Biology and Biotechnology, Maria Curie-Skłodowska University, Akademicka 19, 20-033 Lublin, Poland; roman.paduch@mail.umcs.pl (R.P.)
[3] Department of General and Pediatric Ophthalmology, Medical University of Lublin, Chmielna 1 Street, 20-079 Lublin, Poland (R.P.)
[4] Department of Organic Chemistry, Medical University of Gdańsk, Al. Gen. J. Hallera 107, 80-416 Gdańsk, Poland; krzysztof.szafranski@gumed.edu.pl (K.Szafrański)
[5] Department of Industrial and Environmental Microbiology, Institute of Biological Sciences, Faculty of Biology and Biotechnology, Maria Curie-Skłodowska University, Akademicka 19, 20-033 Lublin, Poland; adrian.wiater@mail.umcs..pl (A.W.)
* Correspondence: roman.paduch@mail.umcs.pl; adrian.wiater@mail.umcs.pl

Contents:

Tables for In Vitro Evaluation of Cytotoxicity, Apoptosis Induction and Antioxidant Activity of CM-1 – CM-4 p. 2-3

Figures for ^{1}H, ^{13}C and ^{31}P NMR spectra of CM-1 – CM-4 p. 4-8

High-resolution mass spectra (HRMS) for CM-2 and CM-4 p. 9-10

In Vitro Evaluation of Cytotoxicity, Apoptosis Induction and Antioxidant Activity of CM-1 – CM-4

Table S1. Statistical analysis of the percentages of HT29 and CCD 841 CoTr cells in the sub-G1; G1; S and G2 phases after 24 h incubation with 200 μg/mL concentration of tested coumarins

Cell cycle phase	Control	HT29			
		**CM-3**	**CM-4**	**CM-2**	**CM-1**
Sub-G1	1.33±0.11	1.71±0.06	2.22±0.17*	3.11±0.14*	3.82±0.13*
G1	50.04±0.54	70.76±0.86*	67.35±0.65*	48.21±0.43	52.17±0.71
S	15.61±0.36	2.79±0.07*	1.53±0.11*	18.74±0.19	17.73±0.47
G2	29.47±0.27	23.68±0.93	28.21±0.62	26.70±0.32	23.56±0.37
		**CCD 841 CoTr**			
		**CM-3**	**CM-4**	**CM-2**	**CM-1**
Sub-G1	17.49±1.08	16.92±0.68	21.96±0.27*	19.25±0.31*	30.17±0.14*
G1	30.28±1.93	32.79±0.55	31.92±0.53	30.42±0.18	26.16±0.29*
S	24.45±1.05	19.70±1.28*	19.66±0.12*	22.84±0.38	22.20±1.17
G2	22.74±1.28	25.40±1.00	22.29±0.49	23.22±0.54	22.20±1.82

Table S2. Statistical analysis of the percentages of human colon tumor HT29 being viable and distributed in different stages of cell death (early or late apoptosis and necrosis) after 24 h incubation with 200 μg/mL concentration of tested coumarins.

Cell viability status	Cell culture				
	**HT29**				
	Control	**CM-3**	**CM-4**	**CM-2**	**CM-1**
Viable	95.16±0.57	92.93±0.48	83.54±0.54*	94.85±0.36	92.21±0.30
Early apoptosis	1.10±0.29	1.58±0.10	3.10±0.29*	1.35±0.13	1.89±0.09
Late apoptosis	3.03±0.71	4.67±0.50	11.17±0.62*	3.61±0.17	5.48±0.40
Necrosis	0.72±0.28	0.83±0.08	2.19±0.28*	0.20±0.08*	0.42±0.19

Table S3. Statistical analysis of the percentages of human colon normal cells CCD 841 CoTr being viable and distributed in different stages of cell death (early or late apoptosis and necrosis) after 24 h incubation with 200 μg/mL concentration of tested coumarins.

Cell viability status	Cell culture				
	**CCD 841 CoTr**				
	Control	**CM-3**	**CM-4**	**CM-2**	**CM-1**
Viable	92.32±0.52	91.39±0.85	83.68±0.65*	90.31±0.91	87.46±0.06*
Early apoptosis	1.81±0.21	1.81±0.44	1.99±0.30	2.82±0.70	3.62±0.24*
Late apoptosis	5.61±0.51	6.21±0.68	13.32±0.89*	6.03±0.54	7.86±0.15*
Necrosis	0.28±0.04	0.59±0.26	1.02±0.54*	0.85±0.30*	1.06±0.12*

Table S4. DPPH free radicals scavenging activity (%). The % of reduced DPPH. Radical by tested coumarins is compared to the control (0% of reduction).

Coumarin	Coumarin concentration (µg/mL)	Reduction value, which correspond to the following Trolox concentration (µg/mL)
**CM-1**	25	0
	100	1.444±0.131
	200	3.162±0.061
**CM-2**	25	8.142±0.331
	100	17.721±0.316
	200	25.343±0.131
**CM-3**	25	2.786±0.205
	100	8.349±0.417
	200	12.454±0.436
**CM-4**	25	3.528±0.307
	100	9.320±0.090
	200	12.995±0.120

Table S5. Ferric-reducing antioxidant power assay (FRAP). The value of conversed Fe(III) to Fe(II) by tested coumarins is compared to the control (0% of reduction). As control, methanol anhydrous, 99.8% was used. The results are presented as reduction values (µg/mL) corresponding to the appropriate concentration of ascorbic acid.

Coumarin	Coumarin concentration (µg/mL)	Reduction value, which correspond to the following trolox concentration (µg/mL)
**CM-1**	25	0.227±0.202
	100	2.790±0.755
	200	7.143±0.380
**CM-2**	25	4.111±0.378
	100	4.679±0.618
	200	9.046±0.730
**CM-3**	25	0.906±0.614
	100	1.471±0.316
	200	2.035±0.435
**CM-4**	25	1.847±0.377
	100	3.923±1.070
	200	7.903±0.381

^{1}H, ^{13}C and ^{31}P NMR spectra of CM-2 – CM-4

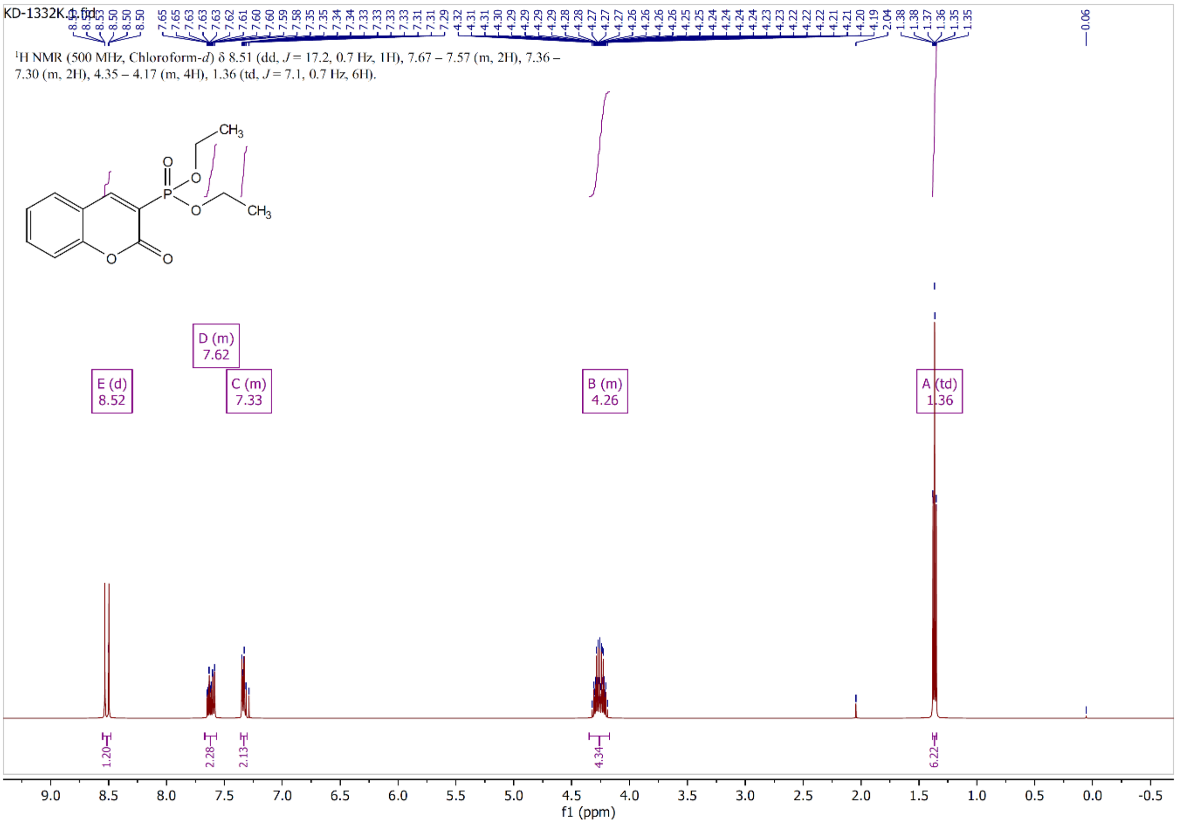

Figure S1. ^{1}H NMR of diethyl (2-oxo-2H-chromen-3-yl)phosphonate (CM-2).

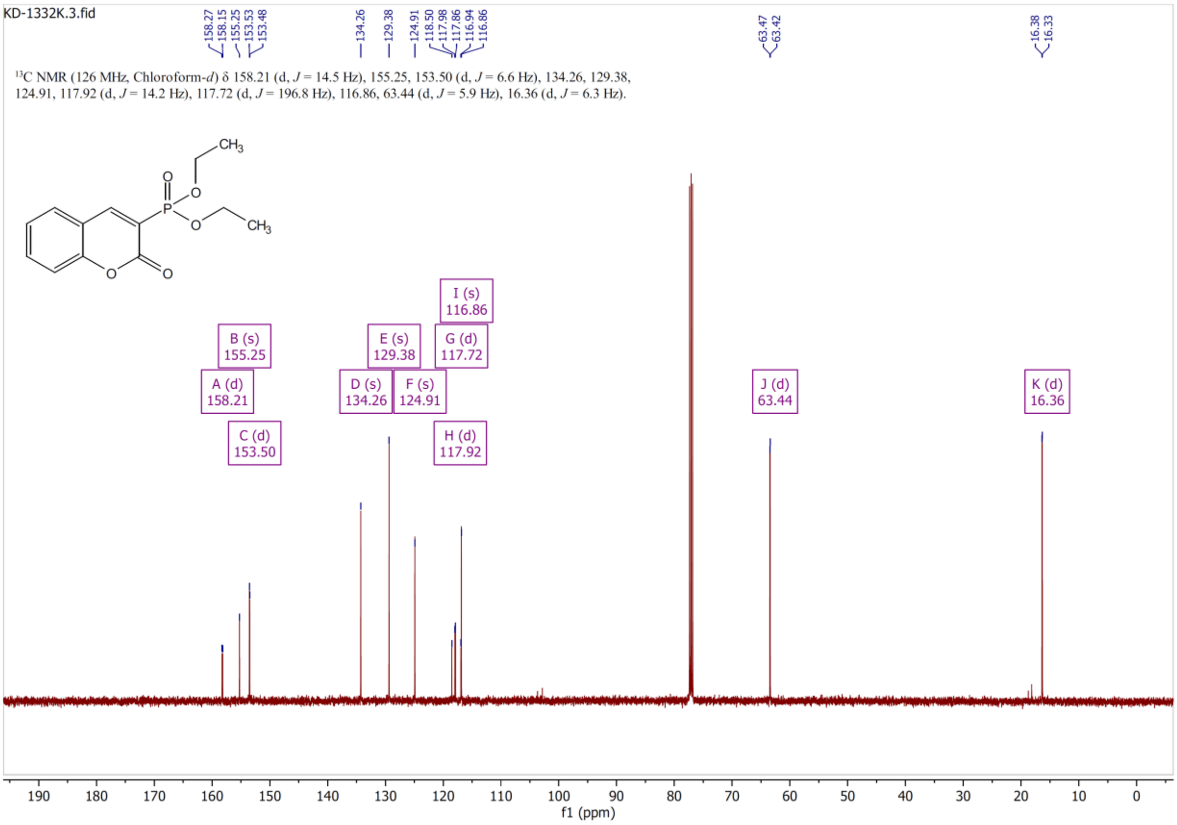

Figure S2. ^{13}C NMR of diethyl (2-oxo-2H-chromen-3-yl)phosphonate (CM-2).

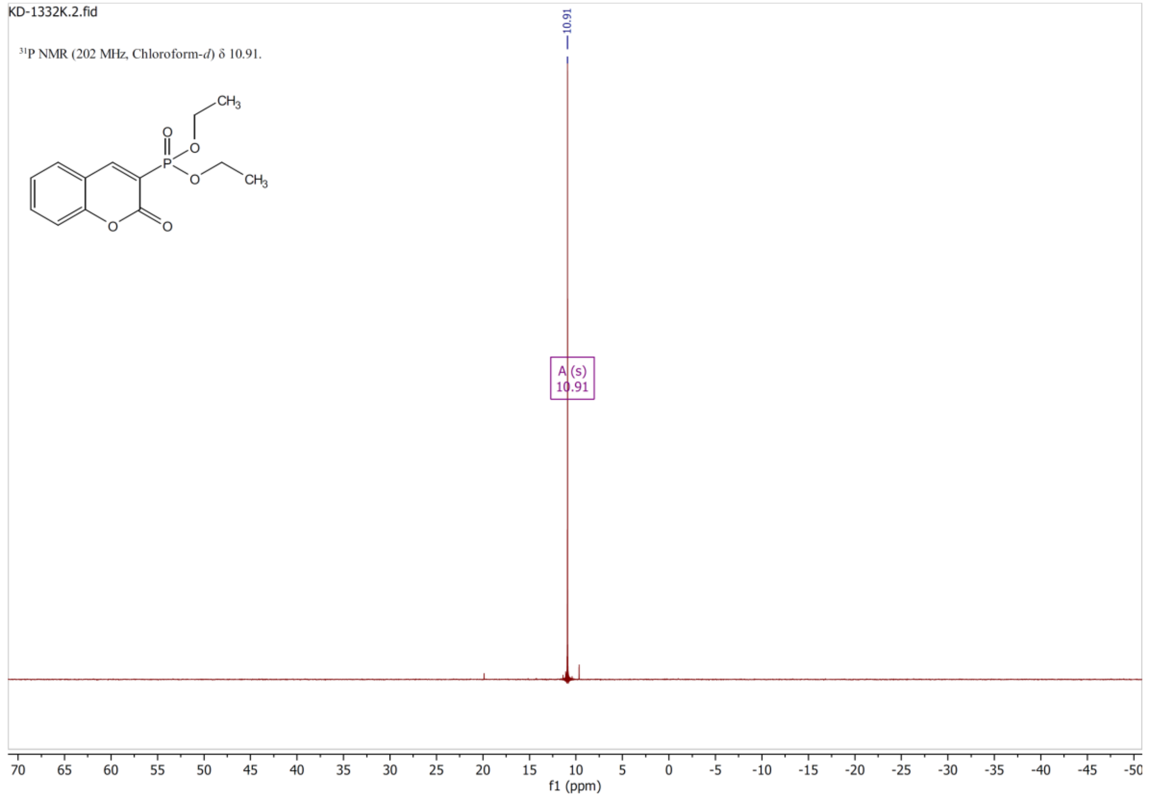

Figure S3. ³¹P NMR of diethyl (2-oxo-2H-chromen-3-yl)phosphonate (CM-2).

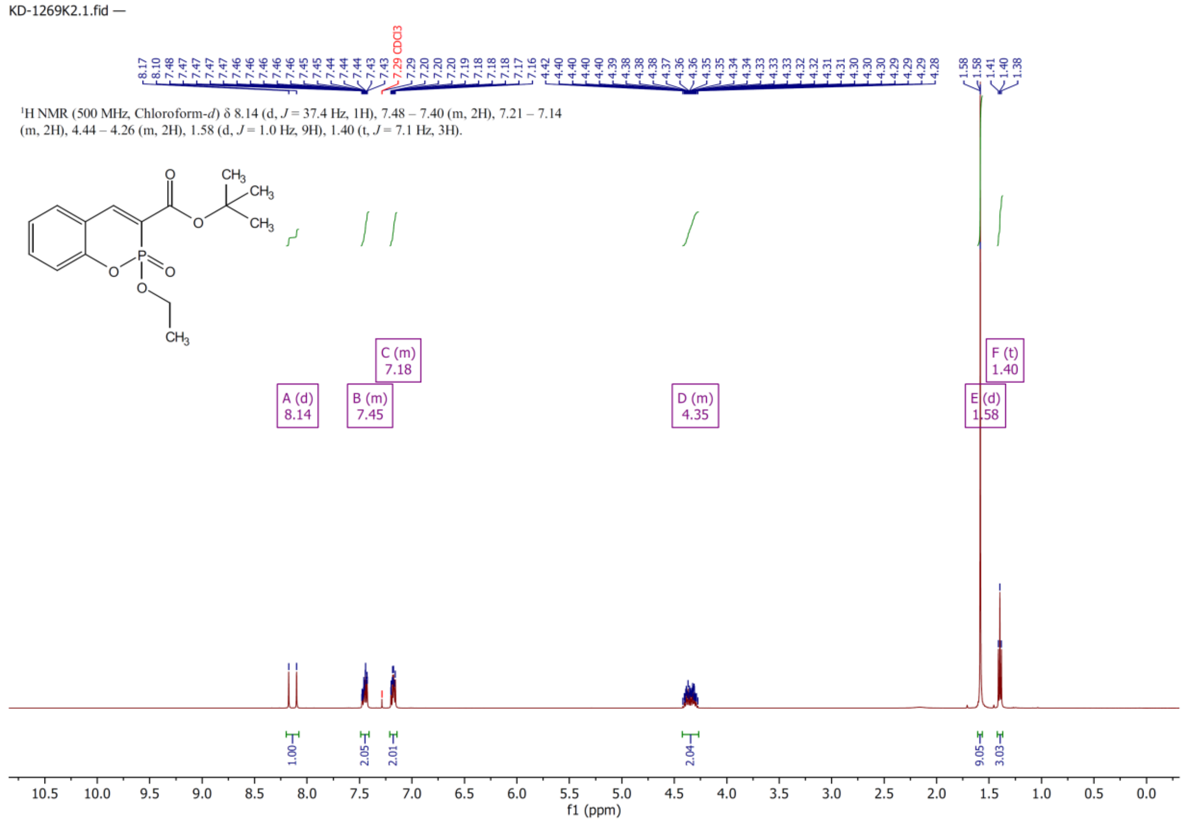

Figure S4. ¹H NMR of *t*-Butyl (2-ethoxy-2-oxo-2H-1,2-benzoxaphosphorine)-3-carboxylate (CM-3).

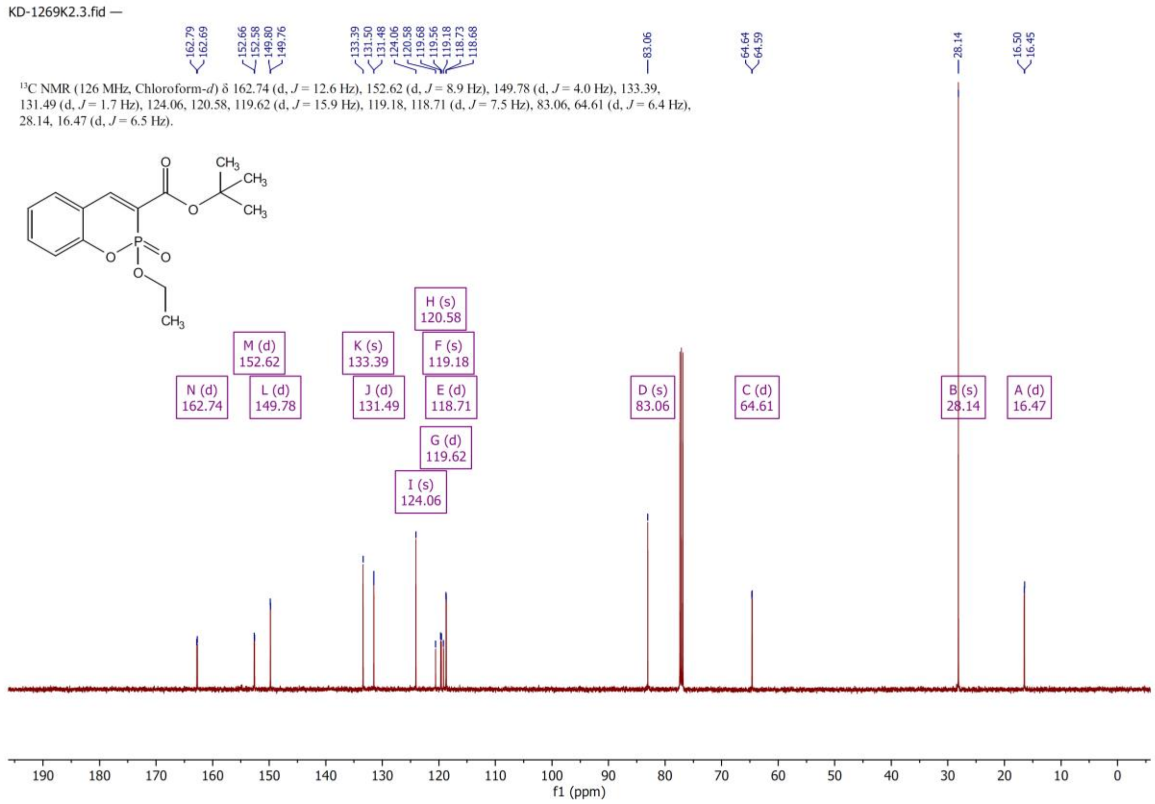

Figure S5. ^{13}C NMR of *t*-Butyl (2-ethoxy-2-oxo-2H-1,2-benzoxaphosphorine)-3-carboxylate (CM-3).

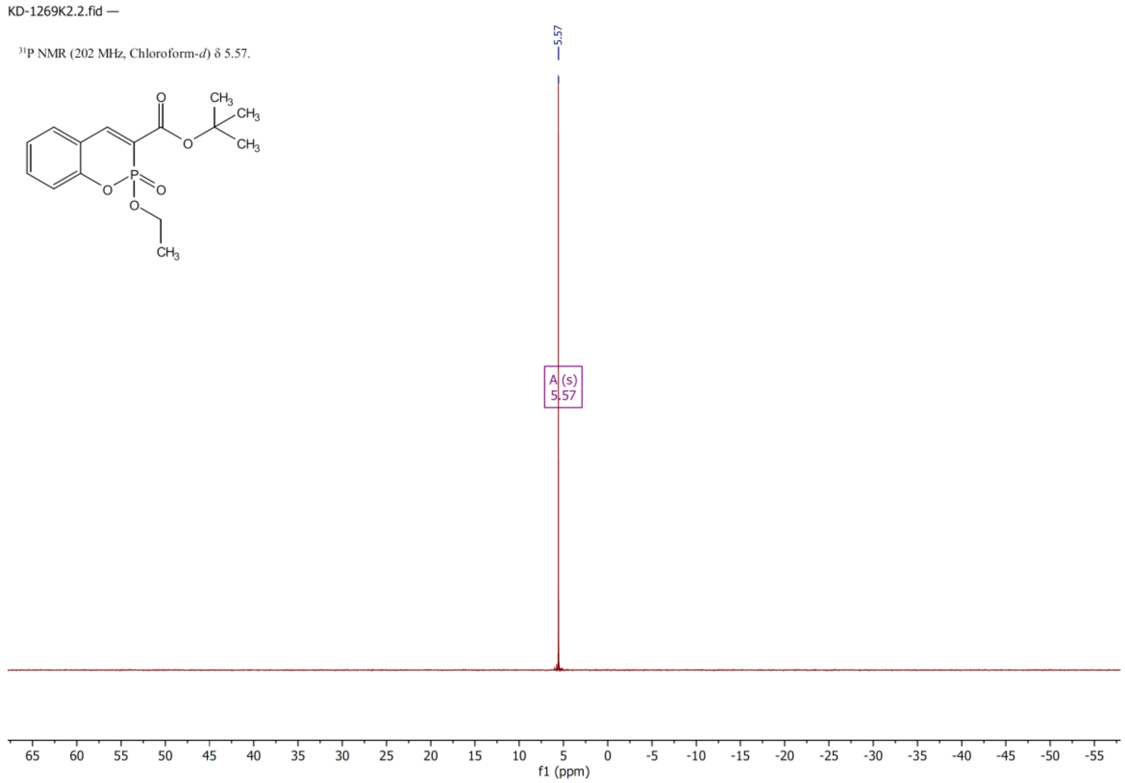

Figure S6. ^{31}P NMR of *t*-Butyl (2-ethoxy-2-oxo-2H-1,2-benzoxaphosphorine)-3-carboxylate (CM-3).

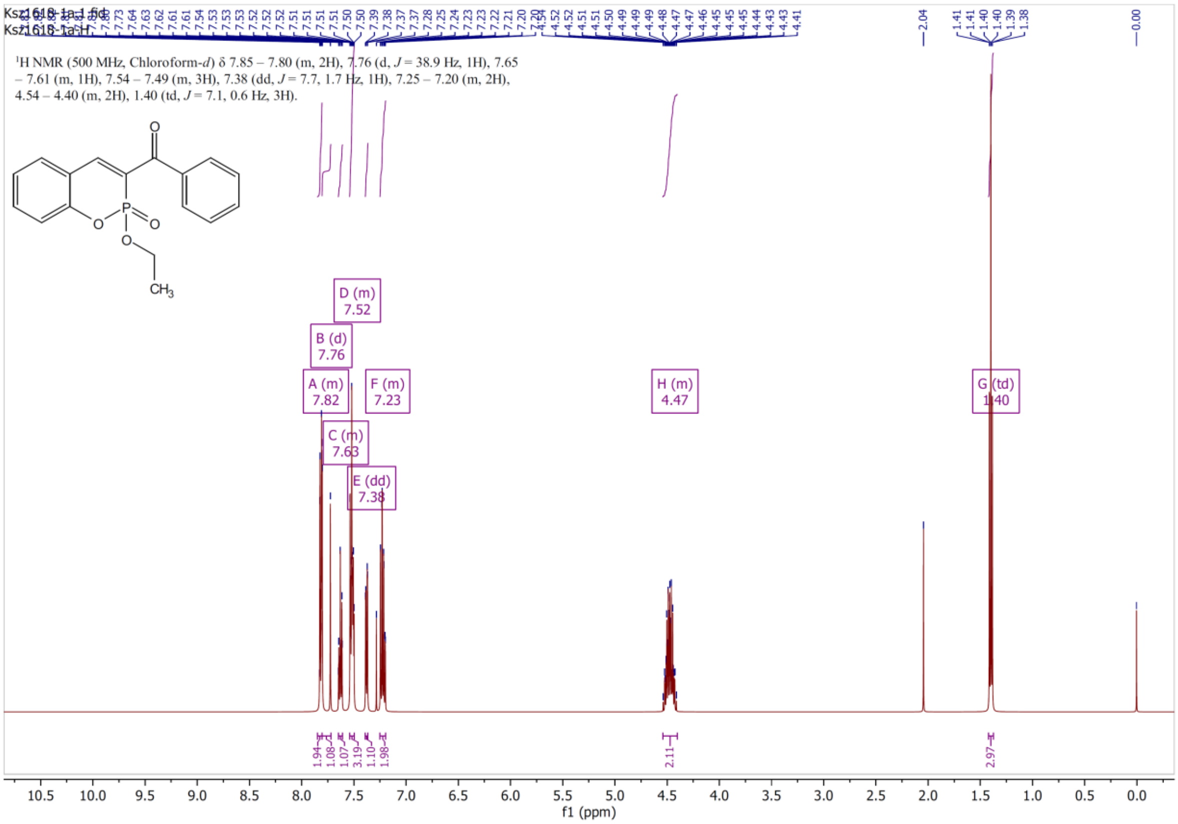

Figure S7. ¹H NMR of 3-benzoyl-(2-ethoxy-2-oxo-2H-1,2-benzoxaphosphorine) (CM-4).

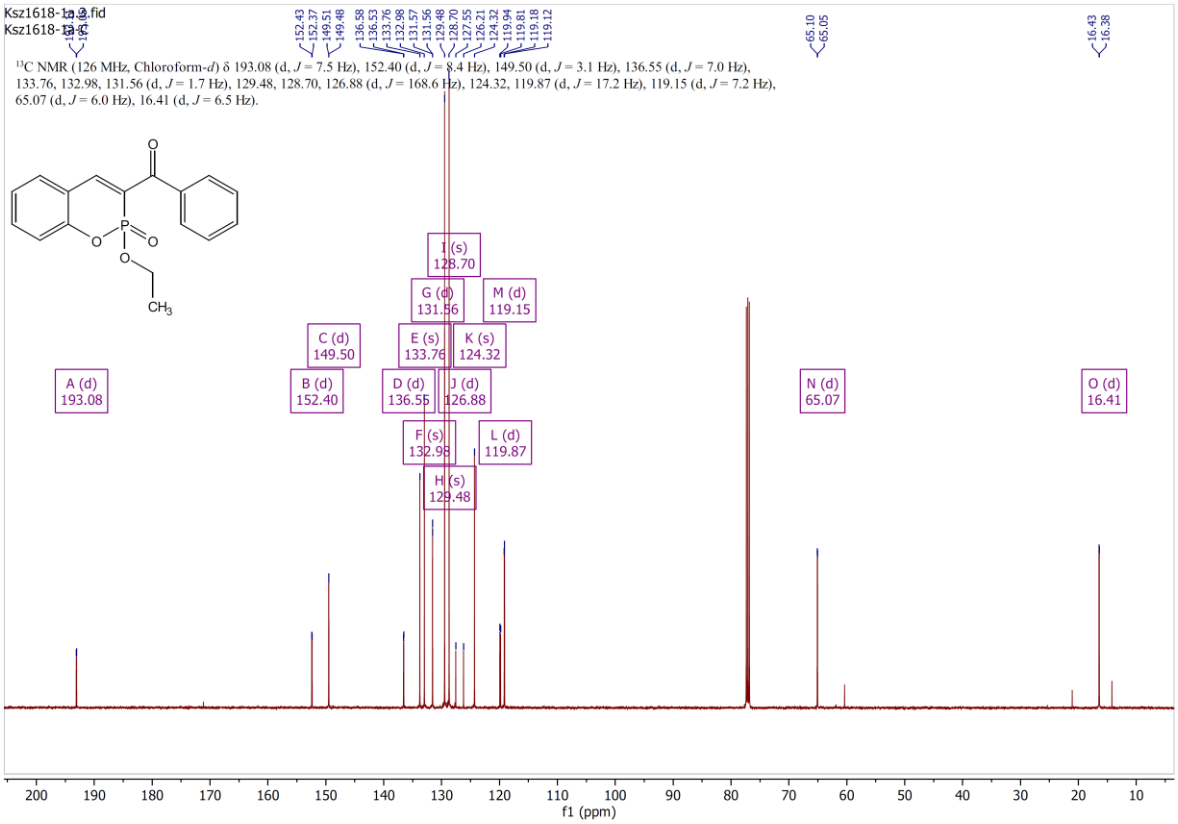

Figure S8. ¹³C NMR of 3-benzoyl-(2-ethoxy-2-oxo-2H-1,2-benzoxaphosphorine) (CM-4).

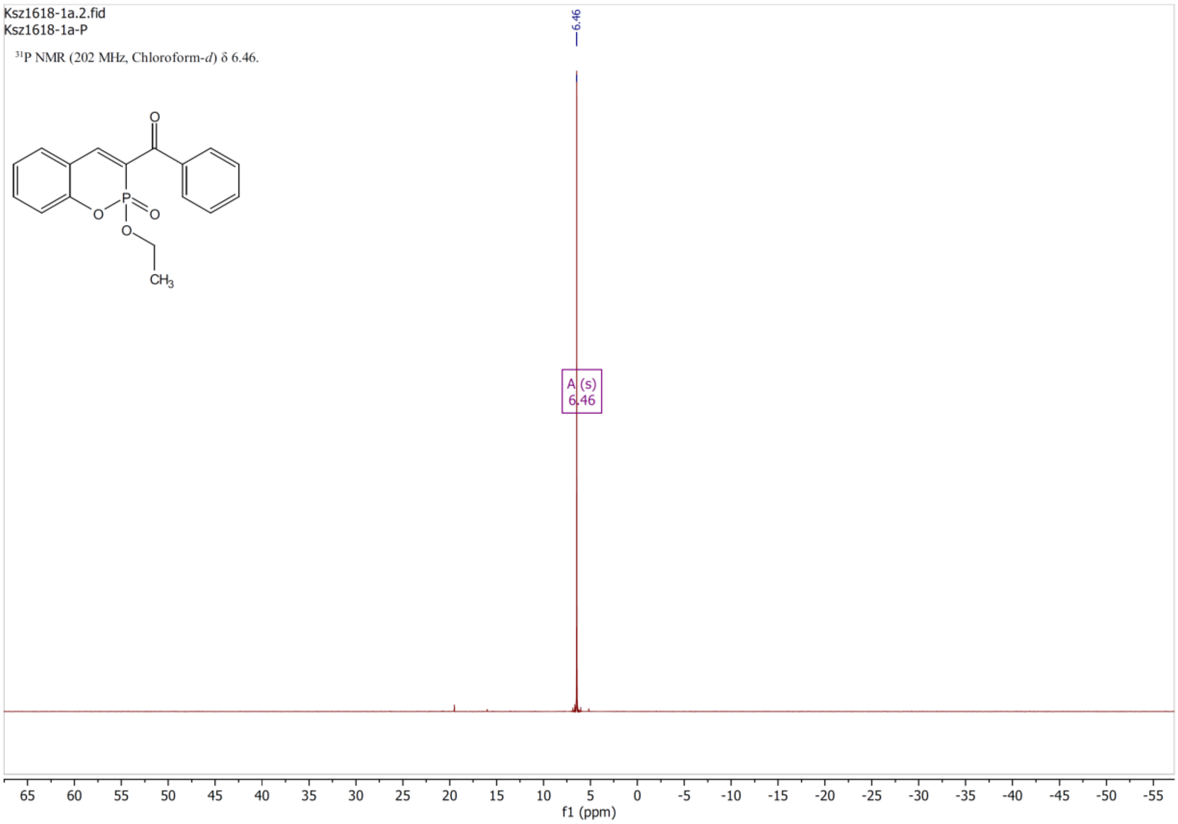

Figure S9. ^{31}P NMR of 3-benzoyl-(2-ethoxy-2-oxo-2H-1,2-benzoxaphosphorine) (CM-4).

High-resolution mass spectra (HRMS) for CM-2

Acquired by	: Admin
Sample Name	: KSz-1575-3
Sample ID	:
Vail #	: 12
Injection Volume	: 0.1 uL
Data File Name	: KSz-1575-3_CH3CN_H2O-5-30min_2021-07-19_4.lcd
Method File Name	: CH3CN_H2O-5-30min.lcm
Batch File Name	: 2021-07-19.lcb
Report File Name	: DefaultLCMS.lcr
Data Acquired	: 19.07.2021 17:33:55
Data Processed	: 20.07.2021 8:22:05

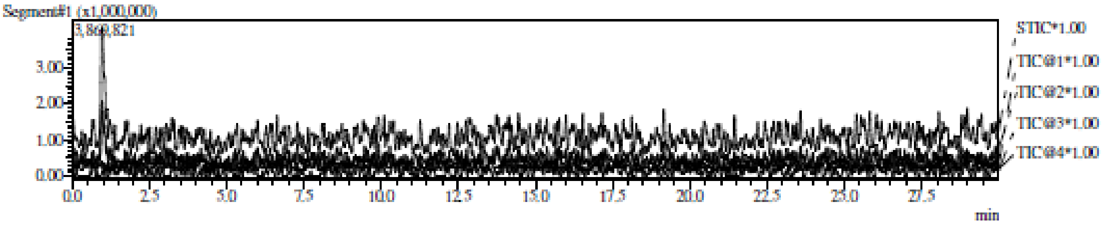

PeakTable

Detector A Ch1 254nm

Peak#	Ret. Time	Area	Height	Area %	Height %
1	0.920	27570	6127	64.560	89.941
2	1.633	1450	141	3.396	2.069
3	2.408	8612	264	20.166	3.870
4	2.769	5072	281	11.878	4.121
Total		42704	6812	100.000	100.000

MS Spectrum Graph

#:1 Ret.Time: Averaged 1.000-1.000(Scan#:402-402)
BG Mode:None
Mass Peaks:3 Base Peak:587.1217(671349) MS Stage:MS Polarity:Pos Segment1 - Event2 Precursor:--- Cutoff: Ionization Mode:E

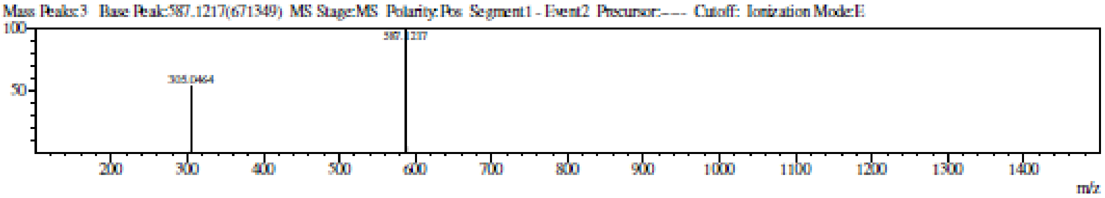

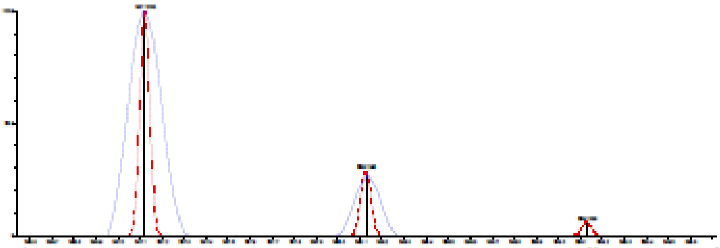

Rank	Score	Formula (M)	Ion	Meas. m/z	Pred. m/z	Diff (mDa)	Diff (ppm)	Iso Score	DBE
1	70.87	C26 H30 O10 P2	[M+Na] +	587.1217	587.1206	1.1	1.87	72.45	13.0
2	45.95	C33 H25 O7 P	[M+Na] +	587.1217	587.1230	-1.3	-2.21	47.38	22.0
3	28.13	C40 H20 O4	[M+Na] +	587.1217	587.1254	-3.7	-6.30	36.54	31.0
4	25.86	C26 H29 O12 P	[M+Na] +	587.1217	587.1289	-7.2	-12.26	76.12	13.0
5	22.54	C29 H24 O12	[M+Na] +	587.1217	587.1160	5.7	9.71	52.53	18.0
6	19.63	C40 H21 O2 P	[M+Na] +	587.1217	587.1171	4.6	7.83	31.81	31.0
7	14.59	C33 H26 O5 P2	[M+Na] +	587.1217	587.1148	6.9	11.75	41.29	22.0
8	12.11	C37 H26 O2 P2	[M+Na] +	587.1217	587.1300	-8.3	-14.14	41.83	26.0
9	11.25	C33 H24 O9	[M+Na] +	587.1217	587.1313	-9.6	-16.35	48.76	22.0
10	4.48	C36 H20 O7	[M+Na] +	587.1217	587.1101	11.6	19.76	32.04	27.0

High-resolution mass spectra (HRMS) for CM-4

Acquired by	: Admin
Sample Name	: KSz-1575-7
Sample ID	:
Vail #	: 14
Injection Volume	: 0.1 uL
Data File Name	: KSz-1575-7_CH3CN_H2O-5-30min_2021-07-19_6.lcd
Method File Name	: CH3CN_H2O-5-30min.lcm
Batch File Name	: 2021-07-19.lcb
Report File Name	: DefaultLCMS.lcr
Data Acquired	: 19.07.2021 18:35:05
Data Processed	: 20.07.2021 8:29:26

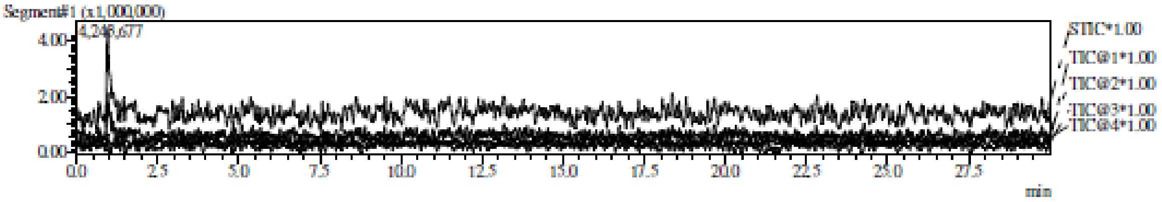

PeakTable

Detector A Ch1 254nm

Peak#	Ret. Time	Area	Height	Area %	Height %
1	0.924	91654	18778	96.994	99.507
2	2.354	2841	93	3.006	0.493
Total		94495	18871	100.000	100.000

MS Spectrum Graph

#:1 Ret.Time: Averaged 0.960-0.960(Scan#:385-385)
BG Mode:None
Mass Peaks:4 Base Peak:337.0517(689437) MS Stage:MS Polarity:Pos Segment1 - Event1 Precursor:--- Cutoff: Ionization Mode:E

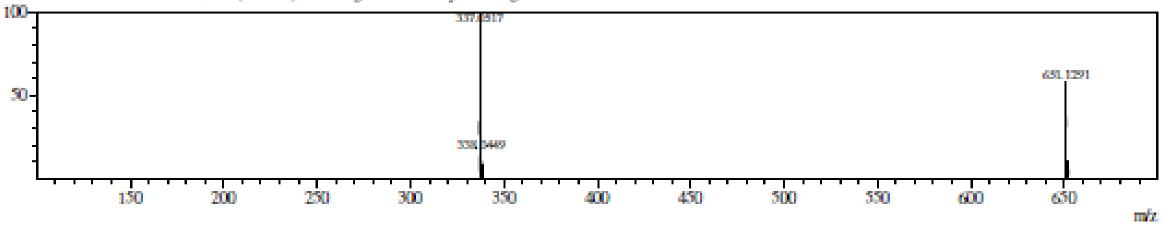

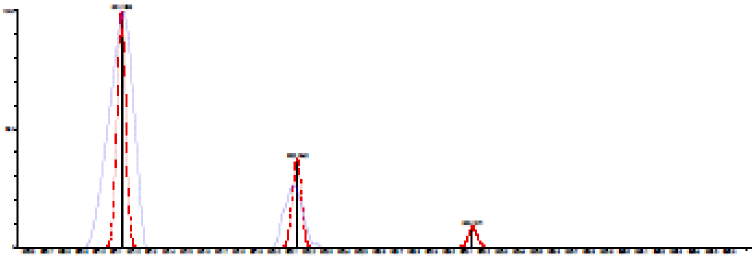

Rank	Score	Formula (M)	Ion	Meas. m/z	Pred. m/z	Diff (mDa)	Diff (ppm)	Iso Score	DBE
1	34.57	C34 H30 O8 P2	[M+Na] +	651.1291	651.1308	-1.7	-2.61	36.02	21.0
2	31.12	C37 H24 O10	[M+Na] +	651.1291	651.1262	2.9	4.45	34.06	26.0
3	22.45	C41 H26 O3 P2	[M+Na] +	651.1291	651.1249	4.2	6.45	29.74	30.0
4	19.30	C41 H25 O5 P	[M+Na] +	651.1291	651.1332	-4.1	-6.30	25.07	30.0
5	7.56	C34 H29 O10 P	[M+Na] +	651.1291	651.1391	-10.0	-15.36	29.40	21.0
6	7.08	C37 H25 O8 P	[M+Na] +	651.1291	651.1179	11.2	17.20	34.02	26.0
7	4.18	C45 H26 P2	[M+Na] +	651.1291	651.1402	-11.1	-17.05	19.74	34.0
8	0.00	C48 H21 P	[M+Na] +	651.1291	651.1273	1.8	2.76	0.00	39.0
9	0.00	C48 H20 O2	[M+Na] +	651.1291	651.1356	-6.5	-9.98	0.00	39.0
10	0.00	C44 H20 O5	[M+Na] +	651.1291	651.1203	8.8	13.51	0.00	35.0
